# Assessing the Potential Prognostic and Immunological Role of TK1 in Prostate Cancer

**DOI:** 10.3389/fgene.2022.778850

**Published:** 2022-04-26

**Authors:** Hui Xie, Linpei Guo, Zhun Wang, Shuanghe Peng, Qianwang Ma, Zhao Yang, Zhiqun Shang, Yuanjie Niu

**Affiliations:** ^1^ Department of Urology, Tianjin Institute of Urology, the Second Hospital of Tianjin Medical University, Tianjin, China; ^2^ Department of Urology, the Affiliated Wuxi No. 2 People’s Hospital of Nanjing Medical University, Wuxi, China; ^3^ Department of Pathology, Tianjin Institute of Urology, the Second Hospital of Tianjin Medical University, Tianjin, China

**Keywords:** thymidine kinase 1, bioinformatics, prognostic biomarker, tumor immunity, prostate cancer

## Abstract

**Background:** It has been reported that thymidine kinase 1 (TK1) was up-regulated in multiple malignancies and participated in the regulation of tumor malignant behavior. However, its specific role in prostate cancer (PCa) remains unclear.

**Methods:** TK1 expression in PCa patients and cell lines was identified via crossover analysis of the public datasets. A series of *in vitro* experiments and *in vivo* models was applied to investigate the function of TK1 in PCa. Functional enrichment analyses were further conducted to explore the underlying mechanism. Additionally, TISIDB was applied to explore the correlation between TK1 expression and tumor-infiltrating lymphocytes, immune subtypes, and immune regulatory factors.

**Results:** TK1 expression was significantly up-regulated in PCa patients and cell lines. TK1 ablation inhibited tumor cell proliferation and migration potential, and *in vivo* experiments showed that TK1 inactivation can significantly restrain tumor growth. Functional enrichment analysis revealed TK1-related hub genes (AURKB, CCNB2, CDC20, CDCA5, CDK1, CENPA, CENPM, KIF2C, NDC80, NUF2, PLK1, SKA1, SPC25, ZWINT), and found that TK1 was closely involved in the regulation of cell cycle. Moreover, elevated mRNA expression of TK1 was related with higher Gleason score, higher clinical stage, higher pathological stage, higher lymph node stage, shorter overall survival, and DFS in PCa patients. Particularly, TK1 represented attenuated expression in C3 PCa and was related with infiltration of CD4^+^, CD8^+^ T cells, and dendritic cells as well as immunomodulator expression.

**Conclusion:** Our study indicates that TK1 is a prognostic predictor correlated with poor outcomes of PCa patients, and for the first time represented that TK1 can promote the progression of PCa. Therefore, TK1 may be a potential diagnostic and prognostic biomarker, as well as a therapeutic target for PCa.

## Introduction

According to the latest American Cancer Society’s statistics, prostate cancer (PCa) ranks first among estimated new cases and second in the number of estimated deaths (Siegel et al., 2020). Furthermore, with an estimated nearly 1.4 million new cases and 375,000 deaths worldwide, PCa is the second most frequent cancer and the fifth leading cause of cancer death among men in 2020 (Sung et al., 2021). At present, fully curative treatment still has not been found for the terminal stage of PCa, castration resistant prostate cancer (CRPC) (Wong et al., 2014). Meanwhile, numerous microarray and next-generation sequencing technologies have been applied to explore the etiology of PCa and to find the specific drug targets (Barbieri et al., 2017). Although important insights have been gained through the efforts, the underlying mechanisms are still not fully clarified. Cumulative evidence suggested that the carcinogenesis and development of PCa is a process involving multiple genes and signaling pathways (Taylor et al., 2010; Grasso et al., 2012; Latonen et al., 2018). Therefore, it is urgent to determine effective molecules to better perform PCa management.

Thymidine kinase 1 (TK1) is a cytosolic enzyme involved in pyrimidine metabolism that catalyzes the addition of a gamma-phosphate group to thymidine and in regenerating thymidine for DNA synthesis and DNA damage (Malvi et al., 2019; Bitter et al., 2020). Among the four deoxyribonucleoside-specific kinases in mammalian cells, TK1 is the only one with the most restricted substrates specificity (Eriksson et al., 2002). Its expression is S-phase dependent and elevated expression of TK1 has been noted in cell proliferation. Since Ki67 is present in all phases of the cell cycle and PCNA is mainly present in later G1, TK1 is more informative because it peaks in S phase expression, closely mimicking the rate of DNA synthesis (Bitter et al., 2020). Recently, it has been applied as an important biomarker for the diagnosis of various cancers, including breast cancer, esophageal cancer, and lung cancer (Li et al., 2005; He et al., 2010; Nisman et al., 2014; Jagarlamudi et al., 2015; Weagel et al., 2018; Malvi et al., 2019). TK1 upregulation was indicated as an early event in a study of breast cancer and further studies demonstrated a positive correlation between TK1 and cancer stage (He et al., 2010; Alegre et al., 2012). Subsequent studies support the potential of utilizing TK1 clinically to identify treatment effectiveness, cancer stage, and prognoses (Nisman et al., 2014; McCartney et al., 2019). Nisman et al. demonstrated that increased serum TK1 levels after chemotherapy for NSCLS indicate treatment failure and poor overall survival (Nisman et al., 2014). As for PCa, a few studies reported that TK1 can be used as a diagnostic biomarker through bioinformatic analysis and serological TK1 may be a potential proliferating biomarker for early detection (Li et al., 2018; Wang et al., 2018; Jagarlamudi et al., 2019; Song et al., 2019; Wang et al., 2020). Wang et al. identified TK1 as a core gene directly related to the recurrence and prognosis of PCa via bioinformatics analysis in multiple databases (Wang et al., 2020). Jagarlamudi et al. found that serum TK1 protein was significantly higher in patients with PCa than in patients with benign urological conditions and that TK1 protein determinations together with PHI or PSAD could be a valuable tool in PCa management (Jagarlamudi et al., 2019). In addition, Li et al. found that serum TK1 levels correlated with Gleason scores of prostate cancer patients whereas PSA levels did not (Li et al., 2018). However, the specific function of TK1 in PCa and the underlying mechanism are still lacking experimental verification.

In the present research, we first systematically investigated the function of TK1 in PCa *via in vivo* and *in vitro* experiments. Cox regression model analysis revealed that the expression of TK1 is significantly correlated with the pathology of PCa and associated with poor survival. Our study revealed that TK1 may be applied as a potential biomarker for PCa.

## Materials and Methods

### Bioinformatic Analysis

The mRNA expression profiles and clinical data were obtained from the cancer genome atlas (TCGA), Gene Expression Omnibus (GEO), Prostate Cancer Transcriptome Atlas (PCTA), and PRAD-TCGA datasets (Rotinen et al., 2018; Cancer Genome Atlas Research, 2015). The PCTA dataset included 1321 clinical specimens. The PRAD dataset refers to the Prostate Adenocarcinoma (TCGA, TCGA Provisional) dataset and contains 497 PCa samples with fully collected data. GEPIA2 (http://gepia2.cancer-pku.cn/) was used to analyze data from the TCGA dataset (Tang et al., 2019). Most gene expression and clinical data were downloaded from cBioPortal (http://cbioportal.org). Also, two PCa microarray datasets were obtained from NCBI GEO (https://www.ncbi.nlm.nih.gov/geo/) (Edgar et al., 2002): GSE70769 (Ross-Adams et al., 2015) and GSE21032 (Taylor et al., 2010). The status of neoadjuvant therapy was not considered as a criterion when selecting samples for analysis. For the PCa specimen shown in the figures, TK1 antibody (Atlas Antibodies, Cat# CAB004683) and AURKB antibody (Atlas Antibodies, Cat#CAB005862) were applied. Immunohistochemical staining of PCa specimens represented moderate cytoplasmic and nuclear positivity in the Human Protein Atlas database^1^ (Uhlen et al., 2010; Uhlén et al., 2015).

Since co-expressed genes may act synergistically with TK1 to play a similar biological function in PCa, we screened the co-expressed genes via Spearman correlation analysis in the PRAD dataset from the cBioPortal^2^ (Cerami et al., 2012). Then Metascape (https://metascape.org)^3^ (Zhou et al., 2019) was applied to conduct further gene enrichment analysis using positively co-expressed genes (r ≥ 0.7, *p* < 0.01, q < 0.01) and TK1. The protein-protein interaction (PPI) enrichment analyses were explored via The Molecular Complex Detection (MCODE) algorithm.

To investigate the correlation between TK1 expression and gene-level copy number variation, the PRAD dataset from TCGA was obtained from cBioPortal online dataset. TIMER was used to analyze the association between TK1 and tumor immune infiltration, immune subtype of PCa^4^ (Li et al., 2017). TISIDB was used to investigate the expression of TK1 in PCa patients with different immune subtypes, as well as the correlation between tumor immune infiltration and TK1^5^ (Ru et al., 2019).

### Cell Culture and Transfection

7PCa cells applied in all experiments including BPH-1, LNCaP, C4-2, 22RV1, and DU145 were all derived from ATCC and cultured in RPMI 1640 (Gibco, United States) with 10% fetal bovine serum (FBS, Gibco, United States) in 5% CO_2_ at 37°C. TK1 shRNA was used to target TK1 mRNA region (GCA​CAG​AGU​UGA​UGA​GAC​G) following the manufacturer’s instructions.

### RNA Isolation, Reverse Transcription, and Quantitative RT-PCR

TRIzol reagent (Sigma, United States) was applied to conduct RNA extraction. RNA reverse transcription was conducted following the protocol by using RevertAid First Strand cDNA Synthesis Kit (ThermoFisher, United States). Quantitative RT-PCR was performed using Fast SYBR Green Master Mix (Roche, United States) on LightCycler 480 System (Roche). Gene expression levels were identified via the Ct method and further normalized to GAPDH levels. The primer sequences are listed in Supplementary Table S1.

### Western Blot

RIPA buffer was applied to extract total cellular protein. The concentration of the protein was quantified by BCA analysis. Then sodium dodecyl sulfate-polyacrylamide sodium gel electrophoresis (SDS-PAGE) and PVDF membrane (Millipore, Bedford, MA) were used to separate the protein. The PVDF membrane was blocked with 5% skim milk for 1 h and incubated overnight with anti-GAPDH (1:2000, ab8245, Abcam) and anti-TK1 (1:1000, ab76495, Abcam) antibody at 4°C. The next day, the membrane was washed and incubated with HRP-conjugated goat anti-rabbit IgG antibody at room temperature for 1 h. Visualization and photography were performed using immobilon western chemilum hrp substrate (WBKLS0100, Millipore).

### Cell Growth Assay

Cell Counting Kit-8 (CCK8, Dojindo, Japan) was applied to analyze cell viability following the corresponding protocols.

### Transwell

For migration assessment, standard transwell chambers (Corning, United States) were used. There were 1.5 × 10^4^ cells with RPMI 1640 medium added to the upper chamber and the lower chamber and was supplemented with 10% fetal bovine serum medium of a 24-well plate. After incubating for 2 d in 37°C, cells were washed with cool PBS twice, fixed with methanol for 30 min at room temperature, stained with 0.2% crystal violet for 20 min, and observed under microscope. Each experiment was conducted in triplicate and repeated three times.

### Flow Cytometry

The effects of TK1 ablation on PCa cell cycle were explored via flow cytometry (FC5000, BD, United States). There was 1 ug/ml propidium iodide (BD Biosciences, Germany) used to stain cancer cells.

### Colony Formation Assay

First, about 1000 cells were seeded in a 6-well plate. After 10 d incubation, the cells were washed with cold PBS, fixed with 4% paraformaldehyde, and stained with 1% crystal violet solution for 10 min. Then the colonies were counted under an optional microscope.

### Tumor Xenograft

All animal experiments were approved by the ethics committee of the Second Hospital of Tianjin Medical University. Eight-week-old nude mice were obtained from Beijing HFK Bioscience Co. Ltd. (Beijing, China). In brief, a total of 10 mice were randomly allocated to 2 groups, and 2 × 10^6^ TK1 knockdown and control cells were suspended in 0.1 ml PBS and injected subcutaneously into the right groin of nude mice. Then the speed of tumor growth was measured every other day.

### Statistics

All statistical analysis was conducted via R-4.0.0 and SPSS 22.0. The following R package were used: edgeR, WGCNA, survival, and ggplot2. Independent Student t-test and ANOVA were both applied for comparison. The Cox regression was used to explore the prognostic value of TK1 expression for OS, as well as DFS. Survival analysis was calculated and carried out by Kaplan-Meier method, and log-rank test was used to determine the distinctions. The data was demonstrated as mean ± standard deviation (SD). A *p* value < 0.05 was considered statistically significant.

## Results

### Elevated Expression of TK1 in Human Prostate Cancer and Cancer Cells

**FIGURE 1:**
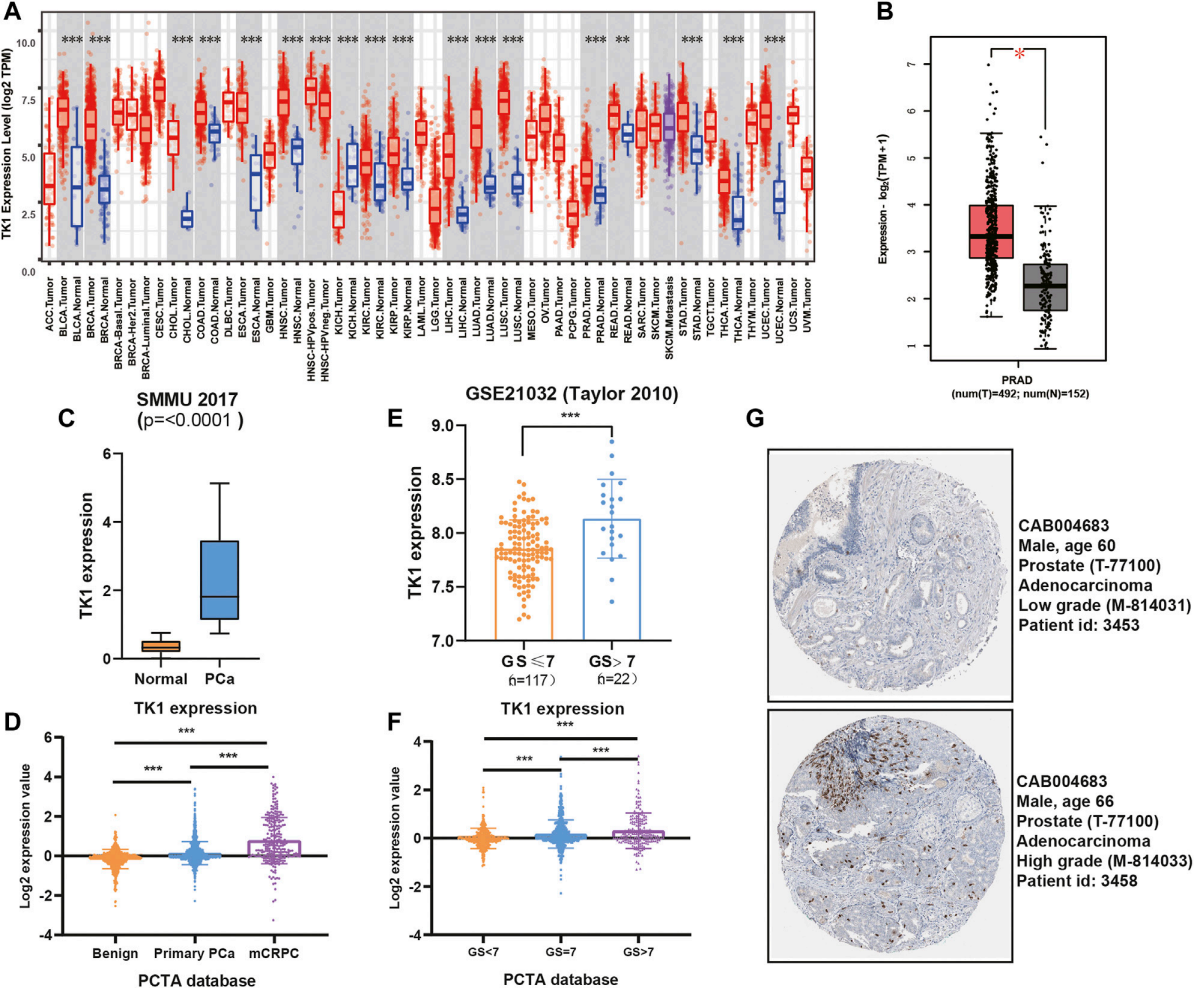
TK1 expression in PCa patients. **(A)** TK1 expression in various cancer tissues and normal tissues. **(B)** TK1 expression in TCGA PRAD cohort. **(C,E)** TK1 expression across several independent clinical studies. **(D,F)** TK1 mRNA expression in the PCTA dataset. **(G)** TK1 protein expression showed by immunohistochemical staining in high-grade and low-grade patient. The pictures were taken from the Human Protein Atlas dataset. *p < 0.05, **p < 0.01, ***p < 0.001; GS, Gleason score; mCRPC, metastatic castration resistant prostate cancer.

Previously studies have reported that TK1 took a key role in tumor initiation and progression. We first explored the expression pattern of TK1 in certain tumors using the TCGA dataset. We found that TK1 was upregulated in most human cancers, including PCa (Figures 1A,B, *p* < 0.05). We also identified elevated expression of TK1 in the Chinese cohort population (Figure 1C) (Ren et al., 2018). Moreover, the expression of TK1 was also elevated in mCRPC patients comparing with primary PCa (Figure 1D). To further assess the expression pattern of TK1 expression in PCa, the correlation between tumor Gleason score and TK1 expression was also explored. As depicted in Figures 1E,F, the expression of TK1 increased with the increase of tumor Gleason score (*p* < 0.001). Next, we explored the TK1 protein expression via The Human Protein Atlas. As showed in Figure 1G, a high-grade PCa patient (ID:3458) showed significantly higher intensity level of TK1 protein expression relative to a low-grade PCa patient (ID:3453).

**FIGURE 2:**
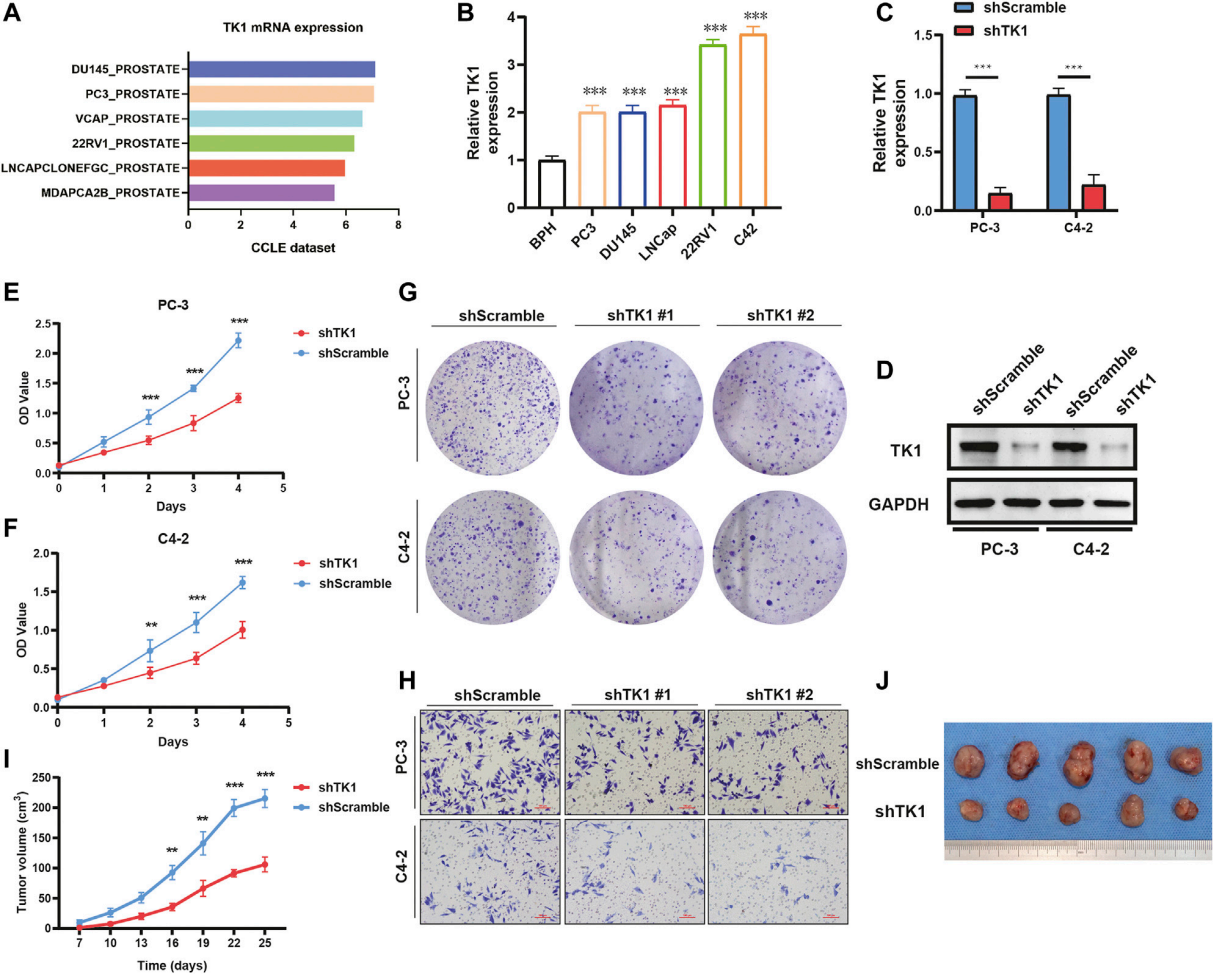
TK1 ablation inhibits tumor cell growth both *in vitro* and *in vivo*. **(A)** TK1 mRNA expression of prostate cancer cell lines in the CCLE dataset. **(B)** TK1 mRNA expression in prostate cancer lines validated by qPCR. **(C,D)** TK1 knockdown efficacy validated by qPCR **(C)** and Western blot **(D)**. **(E,F)** The cell proliferation capacity in shTK1 cells is significantly suppressed compared to control cells. Both PC-3 and C4-2 cell lines were applied. **(G)** TK1 silencing dramatically inhibits the colony formation of prostate cancer cells. **(H)** The migration ability in shTK1 cells is significantly inhibited compared to control cells. **(I)** Tumor growth curves of the TK1-silenced and control groups. **(J)** The photograph of tumors implanted with TK1-silenced PC-3 cells and control tumors from nude mice. **p < 0.01, ***p < 0.001.

To validate the findings in the above datasets, we evaluated the expression of TK1 among multiple human prostate cancer cell lines using the CCLE dataset and quantitative RT-PCR analysis. The data from the CCLE dataset exhibited a certain amount of TK1 expression in PCa cells, and PCR verification demonstrated that its expression was dramatically elevated compared with BPH1 (Figures 2A,B).

### TK1 Inactivation in Prostate Cancer Cells Inhibits Tumor Malignant Behavior

To explore the specific function of TK1 in PCa cells, shRNA-mediated assay was applied to ablate TK1 function. We used shRNA-containing lentiviruses to target TK1 (PC-3 and C4-2) and the knockdown efficacy was verified via qPCR and Western blot (Figures 2C,D). Then all cell lines were tested for their tumor malignant behavior including proliferation, migration, and invasion. As Figures 2E,F show, CCK8 assays were performed to determine the cell proliferation viability, and cells from the shTK1 group grew significantly slower than the control group. Moreover, TK1 ablation also significantly inhibited colony formation and brought about a dramatic reduction in the rate of colony formation (Figure 2G). In addition, transwell assay further revealed the potential stimulative role of TK1 on tumor cell mobility in C42 and PC-3 cells. As depicted in Figure 2H, cells that knocked down TK1 failed to cross over the chambers because of their impaired migration capability. Furthermore, xenograft model assay suggested that knockdown TK1 in PC-3 cells significantly inhibited tumor growth compared with scramble cells (Figures 2I,J). All results indicated that TK1 is closely involved in the malignant behavior of PCa cells.

### Enrichment Analysis and PPI

**FIGURE 3:**
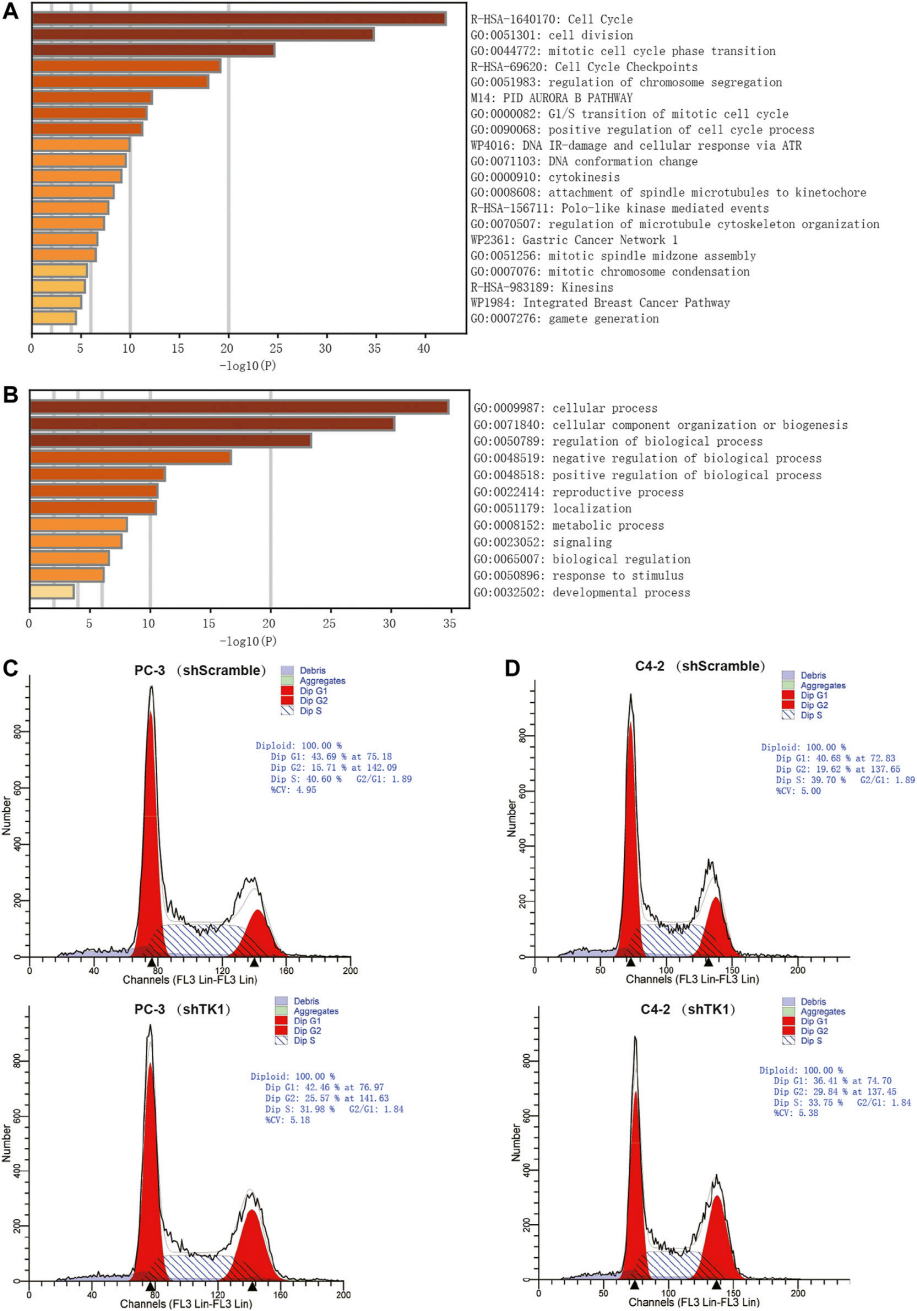
Enrichment analysis and verification of the co-expressed genes. **(A,B)** Bar graph of enriched pathways **(A)** and top-level Gene-Ontology biological processes **(B)** cross the co-expressed genes. **(C,D)** TK1 silencing increases the percentage of cells in the G2/M phase. Cell cycle distributions were investigated by flow cytometry.

To explore the potential biological significance and underlying mechanism of TK1 in PCa, gene co-expression analysis was performed via cBioPortal dataset. Biological functions and related signaling pathways were determined using the top 50 co-expressed genes (r > 0.75, *p*-value <0.05, Table 1). As demonstrated in Figure 3A and Table 2, pathway enrichment analysis revealed the 18 most statistically significant clusters (*p*-value <0.05 and enrichment factor >1.5). Cell cycle, cell division, and chromosome segregation were the top 3 clusters with the most enrichment. Meanwhile, the top-level Gene Ontology biological processes were also demonstrated (Figure 3B). To prove the results of enrichment analysis and further explore the function of TK1 in PCa, cell cycle distributions were identified via flow cytometry. The results indicated that TK1 ablation in prostate cancer cells leads to cell arrest in G2/M phase compared to control cells (Figures 3C,D).

**TABLE 1:** Gene positively correlated with TK1 mRNA expression in the PRAD dataset (Top 50 ranked by Spearman’s correlation coefficient).

Correlated gene	Spearman’s correlation	*p*-Value	q-Value
MCM2	0.857687	3.20E-109	6.42E-105
GINS1	0.837886	1.40E-99	1.41E-95
CDCA5	0.836338	6.99E-99	4.68E-95
KIF2C	0.833796	9.46E-98	4.75E-94
TROAP	0.82942	7.57E-96	3.04E-92
CDC20	0.829119	1.02E-95	3.41E-92
CDC45	0.828222	2.46E-95	7.06E-92
RAD54L	0.826461	1.37E-94	3.43E-91
CHAF1B	0.823953	1.52E-93	3.40E-90
SPC25	0.823389	2.60E-93	5.23E-90
CDT1	0.82312	3.36E-93	6.14E-90
ZWINT	0.822677	5.11E-93	8.56E-90
KIFC1	0.822012	9.58E-93	1.48E-89
NCAPG	0.821661	1.33E-92	1.91E-89
FANCG	0.820582	3.66E-92	4.90E-89
OIP5	0.818721	2.06E-91	2.58E-88
RAD51	0.81848	2.57E-91	3.04E-88
FEN1	0.818403	2.76E-91	3.08E-88
EXO1	0.816745	1.26E-90	1.29E-87
KIF4A	0.816725	1.28E-90	1.29E-87
CDC6	0.816016	2.44E-90	2.34E-87
KIF18B	0.815072	5.74E-90	5.24E-87
CCNB2	0.814834	7.11E-90	6.21E-87
NDC80	0.814465	9.92E-90	8.30E-87
CENPM	0.813052	3.51E-89	2.82E-86
TPX2	0.8123	6.86E-89	5.30E-86
HJURP	0.81152	1.37E-88	1.02E-85
MYBL2	0.810855	2.46E-88	1.76E-85
E2F1	0.810435	3.55E-88	2.46E-85
SKA1	0.810176	4.46E-88	2.92E-85
FANCI	0.810163	4.51E-88	2.92E-85
NUF2	0.808864	1.40E-87	8.79E-85
CENPA	0.807952	3.09E-87	1.88E-84
SKA3	0.807729	3.74E-87	2.21E-84
CDCA3	0.807196	5.93E-87	3.40E-84
FANCD2	0.807027	6.86E-87	3.83E-84
DTL	0.805966	1.70E-86	9.24E-84
MCM10	0.805741	2.06E-86	1.09E-83
TEDC2	0.805711	2.12E-86	1.09E-83
CDK1	0.805485	2.57E-86	1.29E-83
CCNF	0.804815	4.54E-86	2.22E-83
MCM7	0.804701	5.00E-86	2.39E-83
ORC1	0.804614	5.38E-86	2.51E-83
ASF1B	0.802442	3.35E-85	1.53E-82
FAM72B	0.801841	5.54E-85	2.47E-82
PLK1	0.801381	8.13E-85	3.55E-82
PTTG1	0.799776	3.07E-84	1.31E-81
AURKB	0.79901	5.77E-84	2.42E-81
CDC25C	0.798915	6.24E-84	2.56E-81

**TABLE 2:** Top 18 clusters with their representative enriched terms by Metascape.

Category	Description	LogP	Log (q-value)	Symbols
Reactome Gene Sets	Cell cycle	41.0644	−36.706	CDK1, CDC6, CDC20, CDC25C, CENPA, E2F1, FEN1, MCM2, MCM7, MYBL2, ORC1, PLK1, RAD51, CDC45, CCNB2, EXO1, AURKB, PTTG1, GINS1, NDC80, KIF2C, ZWINT, OIP5, TPX2, HJURP, MCM10, SPC25, NCAPG, CENPM, CDT1, NUF2, CDCA5, SKA1, KIFC1
GO Biological Processes	Cell division	30.1137	−26.233	CCNF, CDK1, CDC6, CDC20, CDC25C, CENPA, KIFC1, PLK1, CCNB2, AURKB, PTTG1, NDC80, KIF2C, ZWINT, OIP5, TPX2, KIF4A, SPC25, NCAPG, CDT1, CDCA3, NUF2, CDCA5, KIF18B, SKA1, SKA3, MYBL2
GO Biological Processes	Chromosome segregation	28.9194	−25.164	CDC6, CDC20, FANCD2, FEN1, KIFC1, PLK1, AURKB, PTTG1, NDC80, KIF2C, ZWINT, OIP5, KIF4A, HJURP, SPC25, NCAPG, CDT1, NUF2, CDCA5, KIF18B, SKA1, SKA3, CDC25C, MYBL2, RAD51, RAD54L, CCNB2, TPX2, CCNF, CDK1, E2F1, MCM2, MCM7, ORC1, CDC45, DTL, MCM10, FANCI
WikiPathways	DNA IR-damage and cellular response via ATR	17.8918	−14.813	CDK1, CDC25C, E2F1, FANCD2, FEN1, MCM2, PLK1, RAD51, CDC45, EXO1, FANCI, CDC6, ORC1, DTL, CDT1, MCM7, CCNB2, CENPA, AURKB, MYBL2, TPX2, NCAPG, CCNF
WikiPathways	Cell cycle	17.7783	−14.721	CDK1, CDC6, CDC20, CDC25C, E2F1, MCM2, MCM7, ORC1, PLK1, CDC45, CCNB2, PTTG1, FEN1, RAD51, CHAF1B, EXO1, GINS1, DTL, MCM10, CDT1, FANCG, KIF4A, MYBL2, CDCA5, AURKB
GO Biological Processes	DNA repair	13.5907	−10.876	CDK1, FANCD2, FANCG, FEN1, MCM2, MCM7, RAD51, CHAF1B, CDC45, RAD54L, EXO1, PTTG1, DTL, FANCI, CDCA5, AURKB, E2F1, PLK1
GO Biological Processes	DNA conformation change	12.5687	−9.967	CENPA, MCM2, MCM7, RAD51, CHAF1B, RAD54L, OIP5, HJURP, ASF1B, NCAPG, CENPM, CDCA5, CDC45, CDT1, FEN1
GO Biological Processes	Meiotic cell cycle	10.7912	−8.368	CDC20, CDC25C, FANCD2, PLK1, RAD51, RAD54L, CCNB2, EXO1, PTTG1, NUF2
GO Biological Processes	Positive regulation of cell cycle process	−9.9885	−7.656	CDK1, CDC6, CDC25C, E2F1, FEN1, AURKB, NDC80, DTL, CDT1, CDCA5, ORC1, PLK1, CCNB2, TPX2, KIF18B, CDC20
Canonical Pathways	PID PLK1 pathway	9.74329	−7.423	CDK1, CDC20, CDC25C, PLK1, NDC80, TPX2, CENPA, AURKB, CDT1, CDCA5, CDC6, KIF4A, CCNF, E2F1, KIF2C
Reactome Gene Sets	DNA strand elongation	8.59771	−6.337	FEN1, MCM2, MCM7, CDC45, GINS1, RAD51, FANCD2, RAD54L, CDCA5, EXO1, MCM10
Reactome Gene Sets	Transcriptional regulation by TP53	6.67593	−4.538	CDK1, CDC25C, E2F1, FANCD2, EXO1, AURKB, TPX2, FANCI, CENPA, KIF2C
GO Biological Processes	Positive regulation of chromosome segregation	6.38871	−4.266	CDC6, FEN1, AURKB, CDT1, E2F1, PLK1, HJURP, CDCA5, RAD51
GO Biological Processes	Microtubule polymerization or depolymerization	−5.7257	−3.647	KIF2C, TPX2, KIF18B, SKA1, SKA3, KIFC1, KIF4A, CDK1, PLK1, CCNB2
GO Biological Processes	Gamete generation	5.55847	−3.493	CDC25C, E2F1, FANCD2, FANCG, KIFC1, PLK1, CCNB2, PTTG1, ASF1B
GO Biological Processes	Regulation of microtubule cytoskeleton organization	4.73284	−2.715	CCNF, PLK1, TPX2, SKA1, SKA3
KEGG Pathway	HTLV-I infection	4.08352	−2.118	CDC20, E2F1, MYBL2, CCNB2, PTTG1
GO Biological Processes	Telomere maintenance	3.74684	−1.825	FEN1, RAD51, EXO1, AURKB, DTL

**FIGURE 4:**
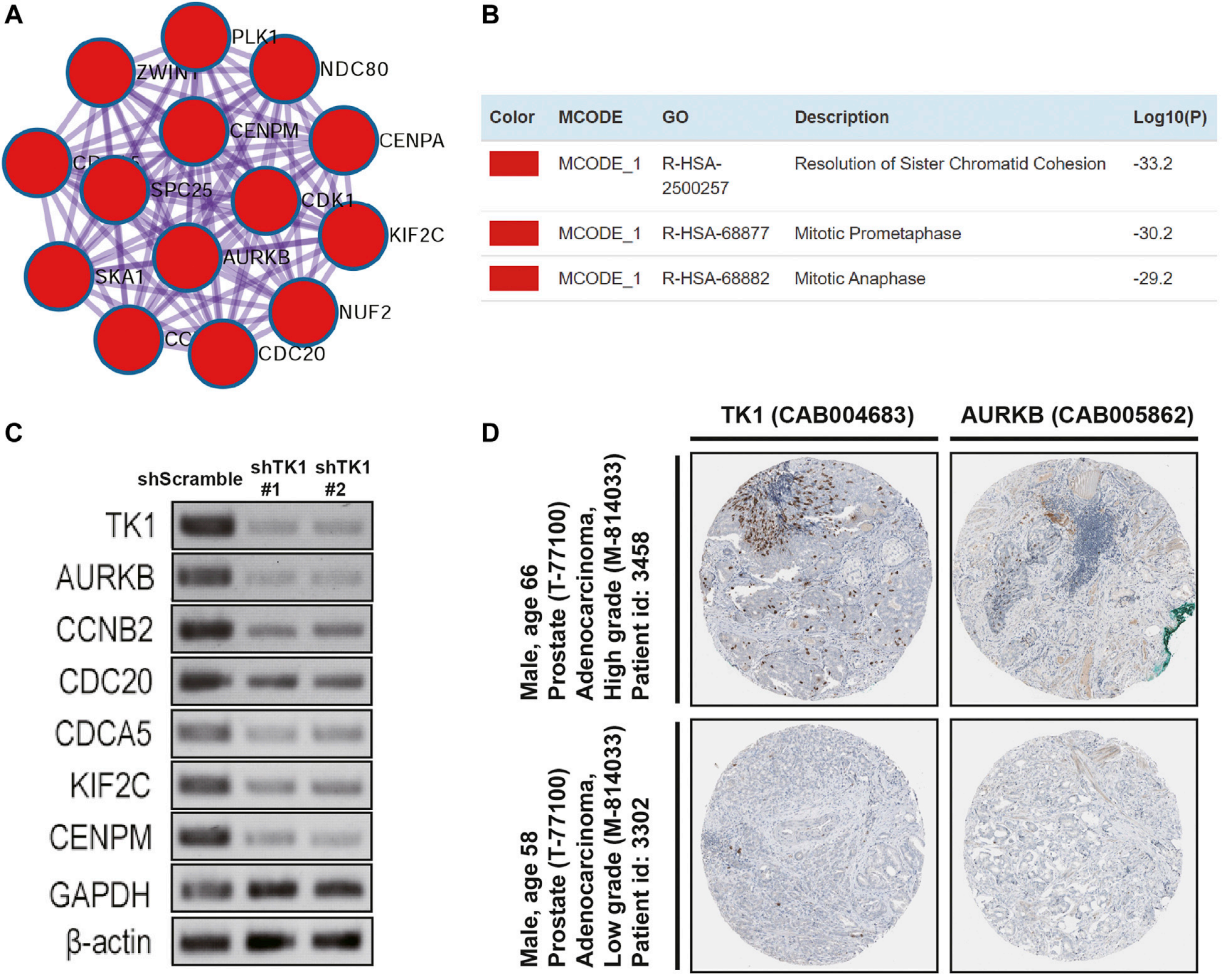
Protein networks and the correlation between TK1 and the hub genes. **(A,B)** Molecular Complex Detection (MCODE) components of the hub genes. **(C)** The expression of serval hub genes was down-regulated in the TK1-silencing cells verified by RT-PCR. **(D)** TK1 and AURKB protein expression showed by immunohistochemical staining in the same high-grade and low-grade patient. The pictures were taken from the Human Protein Atlas dataset.

PPI enrichment analyses were also carried out with the MCODE algorithm to determine densely connected network components. As depicted in Figures 4A,B, the MCODE results were gathered and demonstrated. Fourteen hub genes (AURKB, CCNB2, CDCA5, CDK1, CENPA, CENPM, KIF2C, NDC80, CDC20, NUF2, PLK1, SKA1, SPC25, ZWINT) constituted the MCODE-1 component. The expression relationship between TK1 and all the hub genes was also shown in Supplementary Figure S1, respectively. Moreover, we confirmed that the expression of some hub genes was suppressed in shTK1 cells via RT-PCR (Figure 4C), and the protein co-expression of TK1 and AURKB in a same patient sample in the Human Protein Atlas was depicted in Figure 4D. All the above results indicated that the function of TK1 in PCa was closely involved in cell cycle regulation, which was also in accordance with the phenotypic results characterized previously.

### TK1 Is Correlated With Clinical Features of PCa and Elevated Expression of TK1 Represents a Prognostic Factor for PCa

**FIGURE 5:**
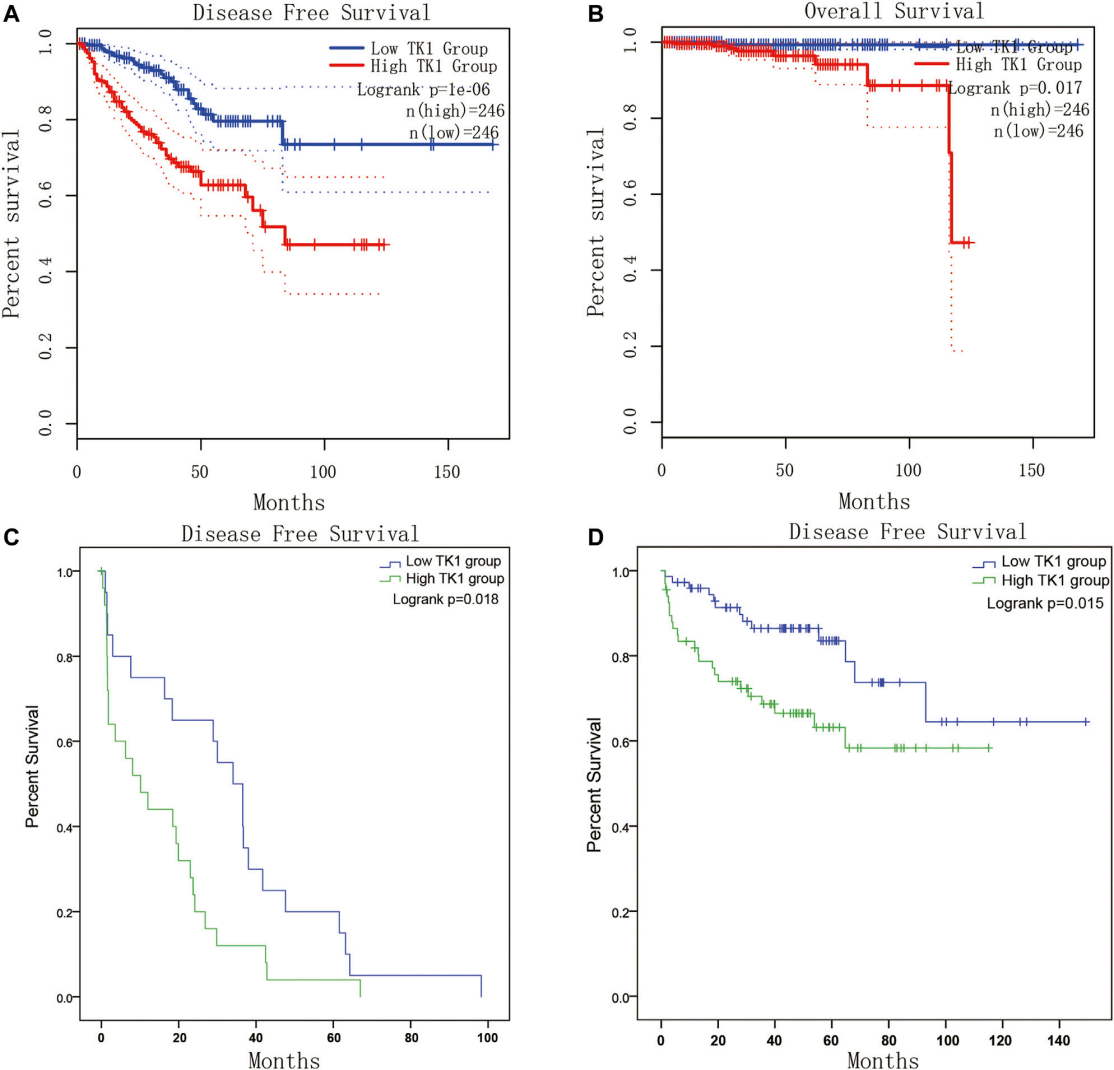
Survival analysis of TK1 expression in PCa. **(A,B)** The TK1 mRNA expression level represented a prognostic value in OS **(A)** and in DFS **(B)** in the PRAD dataset. **(C,D)** Kaplan-Meier plots of the risk of biochemical recurrence in PCa patients with high or low expression of TK1 in several cohorts of human prostate tumors.

Given the crucial capacity of TK1 in PCa, we examined the potential relationship between TK1 expression and clinical features, including multiple clinic-pathological characteristics and survival of PCa patients. Data from the TCGA dataset showed that patients with elder age (>60 years; *p* = 0.003), higher Gleason score (>7; *p* < 0.005), higher clinical stage (≥T3a; *p* < 0.005), higher pathological stage (≥T3a; *p* < 0.001), lymph node metastasis (*p* < 0.005), shorter OS (*p* < 0.005), and shorter DFS (*p* < 0.005) had higher levels of TK1 expression (Table 3). Moreover, the Kaplan-Meier curve method was conducted to determine the correlation between TK1 expression level and OS and DFS (Figures 5A,B). The quartile TK1 mRNA expression level was used as the cutoff point to divided patients into the low TK1 (*n* = 246, TCGA dataset) and high TK1 (*n* = 246, TCGA dataset) group and conducted statistically significant validation of survival analyses in both groups. As Figures 3A,B show, patients in the high TK1 class had a shorter probability of OS (*p* = 0.017) and DFS (*p* < 0.001) compared to the low TK1 group. Moreover, we also investigated the prognostic role of TK1 across several independent clinical data sets (Taylor et al., 2010; Ross-Adams et al., 2015). As depicted in Figures 5C,D, the time to biochemical relapse was significantly shorter in the group of PCa patients with higher TK1 expression.

**TABLE 3:** The correlation between clinicopathological characteristics and TK1 expression in the PRAD dataset.

Characteristics	N	TK1 expression (mean ± SD)	P
Age			0.003
≤60y	224	296.5 ± 306.8	
>60y	275	384.6 ± 345.2	
Clinical stage			<0.001
<T3a	352	328.5 ± 296.3	
≥T3a	55	513.4 ± 557.4	
Pathological stage			<0.001
<T3a	188	253.4 ± 189.5	
≥T3a	304	394.0 ± 340.0	
Gleason score			<0.001
≤7	293	263.7 ± 187.7	
>7	206	460.8 ± 439.6	
Lymph node stage			<0.001
N0	346	324.6 ± 282.4	
N1	80	462.9 ± 379.4	
Overall survival			<0.001
Alive	489	337.4 ± 298.7	
Decease	10	717.8 ± 1034.2	
Disease-free survival			0.001
Disease-free	401	317.2 ± 286.9	
Recurred/progressed	92	435.9 ± 333.6	

To explore the prognostic significance of TK1 in PCa, the Cox regression method was applied. As demonstrated in Table 4, clinical stage (*p* < 0.005), Gleason score (*p* < 0.001), pathological stage (*p* < 0.005), lymph node stage (*p* = 0.014), and TK1 mRNA expression (*p* < 0.001) were suitable to be regarded as prognostic factors for DFS by univariate analysis. In addition, we also found that Gleason score (*p* = 0.007), clinical stage (*p* = 0.01), and TK1 mRNA expression (*p* < 0.001) could be taken as prognostic factors for OS. Furthermore, multi-variate analyses suggested that Gleason score was an independent factor predicting the shortened survival of DFS (*p* < 0.001) and OS (*p* < 0.05), and the clinical stage predicted shorter DFS (*p* = 0.003). Perhaps because of the finite number of deceased in the PRAD dataset, TK1 mRNA expression showed limited prognostic value for survival via multi-variate analysis.

**TABLE 4:** Prognostic value of TK1 mRNA expression level for the disease-free survival (DFS) and overall survival (OS) via Cox proportional model.

	DFS		OS	
	Hazard ratio (95% CI)	P	Hazard ratio (95% CI)	P
Univariate analysis				
Age	1.027 (0.996–1.060)	0.09	1.053 (0.955–1.160)	**0.032**
TK1 mRNA	1.001 (1.000–1.001)	**<0.001**	1.002 (1.001–1.003)	**<0.001**
Clinical stage	1.437 (1.263–1.635)	**<0.001**	1.666 (1.130–2.459)	**0.01**
Pathological stage	1.801 (1.437–2.259)	**<0.001**	1.630 (0.766–3.467)	0.205
Gleason score	2.227 (1.794–2.764)	**<0.001**	2.981 (1.346–6.601)	**0.007**
Lymph node stage	1.831 (1.130–2.969)	**0.014**	3.523 (0.778–15.942)	0.102
Multivariate analysis				
Age	0.997 (0.962–1.034)	0.885	1.041 (0.931–1.163)	0.480
TK1 mRNA	1.000 (1.000–1.001)	0.373	0.999 (0.997–1.002)	0.673
Clinical stage	1.255 (1.079–1.459)	**0.003**	1.278 (0.761–2.145)	0.353
Pathological stage	1.117 (0.794–1.572)	0.525	0.772 (0.255–2.335)	0.647
Gleason score	1.801 (1.310–2.474)	**<0.001**	3.489 (1.035–11.758)	**0.044**
Lymph node stage	0.994 (0.558–1.769)	0.983	2.537 (0.447–14.391)	0.293

The bold values in Table 4 represent values less than 0.05 and are statistically significant.

### Immune Analysis of TK1 in Prostate Cancer

**FIGURE 6:**
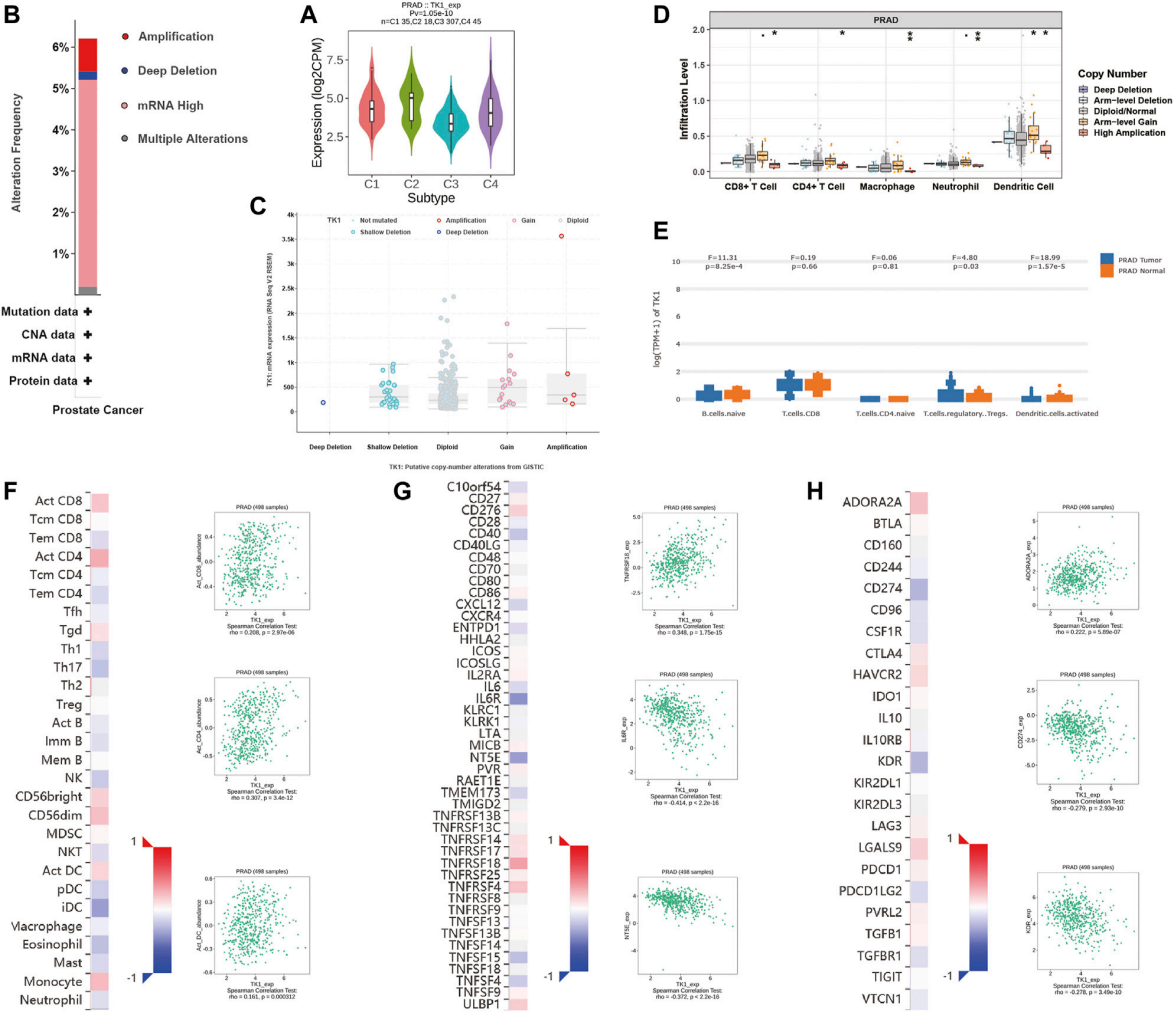
Immune analysis of TK1 in PCa. **(A)** Relationships between TK1 expression and immune subtype in TCGA prostate cancer dataset. **(B)** The mutation types and mutation frequencies of TK1 in PCa. **(C)** Correlation between mRNA expression of TK1 and the copy number in PCa. **(D)** Correlation between TK1 copy number and tumor-infiltrating lymphocytes (TILs). **(E)** TK1 expression of immune cells in the prostate tumor and normal tissues. **(F)** Correlation between TK1 expression and TILs (TISIDB). **(G,H)** Correlation between TK1 expression and immunostimulators **(G)** and immunoinhibitors **(H)**. In the heatmaps of **(F-H)**, the red and blue squares represent positive and negative correlations, respectively. The scatter plots show TILs or immunomodulators with the strongest correlation with TK1 expression.

Next, the correlation between tumor immune infiltration and TK1 expression was analyzed. The results demonstrated that TK1 expression was closely correlated to immune subtypes of PCa, and TK1 was dramatically downregulated in the C3 subtype of PCa (Figure 6A). We further explored the genetic variations of TK1 in 497 cases of PCa in PRAD datasets via cBioPortal. As depicted in Figure 6B, amplification, deletion, and mRNA high were the main genetic variation types in TK1 in all samples. The overall variation rates of TK1 were also represented. In addition, Figure 6C presented the correlation of TK1 mRNA expression and the copy number in PCa. Using TIMER, the correlation between the TK1 copy number and tumor-infiltrating lymphocytes (TILs) was investigated. As shown in Figure 6D, high amplification of TK1 significantly decreased the TILs in PCa (*p* < 0.05). TK1 expression of immune cells in normal tissues and prostate tumor was also shown in Figure 6E. The expression of TK1 in prostate tumors was significantly elevated in Treg cells and decreased in B cells and activated dendritic cells compared with normal tissues (all *p* < 0.05).

We further determined the correlation between TK1 expression, immunomodulators (immunostimulators and immunoinhinitors), and TILs via TISIDB. Figures 6F–H respectively showed the top three TILs and immunomodulators with a Spearman’s correlation coefficient greater than 0.2 with TK1 expression. Activated CD4^+^ (r = 0.307, *p* = 3.4e-12) and CD8^+^ (r = 0.208, *p* = 2.97e-06) T cells depicted the densest association with TK1 (Figure 6F). As depicted in Figures 6G,H, the greatest related immunostimulators with TK1 expression in PCa were interleukin 6 receptor (IL6R, r = −0.414, *p* < 2.2e-16), 5′-nucleotidase ecto (NT5E, r = −0.372, *p* < 2.2e-16), and TNF receptor superfamily member 18 (TNFRSF18, r = 0.348, *p* = 1.75e-15) and the most relevant immunoinhibitors correlated with TK1 expression in PCa were CD274 (r = −0.279, *p* = 2.93e-10), kinase insert domain receptor (KDR, r = −0.278, *p* = 3.49e-10), and adenosine a2a receptor (ADORA2A, r = 0.222, *p* = 5.89e-07).

## Discussion

In this research, we expanded the capacity of TK1, and explored the specific function of TK1 in PCa, as well as its underlying mechanism for the first time. Moreover, we also found that the functions of TK1 were strongly associated with related signaling pathways, including cell cycle, cell division, and mitotic cell cycle phase transition, thereby promoting tumor malignant behavior.

TK1 is a cytosolic enzyme involved in salvage pathway and plays a vital role in pyrimidine deoxynucleotide synthesis during the cell cycle. Thymidine is transferred from the extracellular space to the cell membrane by facilitated diffusion and is converted to the monophosphate form (dTMP) by TK1 at the cell membrane (Bello, 1974; Johnson et al., 1982). In addition to DNA synthesis, TK1 is also essential for cell repair following DNA damage due to its vital role in nucleotides formation beyond the S phase (Chen et al., 2010; Jagarlamudi and Shaw, 2018). The expression level of TK1 increases significantly after cellular damage caused by radiation or chemotherapeutic agents, and depletion of TK1 in cells exposed to DNA damage can lead to cell death (Chen et al., 2010; Fischer et al., 2016; Jagarlamudi and Shaw, 2018). Multiple studies have reported that regulation of cell cycle factors, including TK1, is critical for cell homeostasis and that mutations or dysregulation of cell cycle proteins is a major cause of tumorigenesis (Collins et al., 1997; Levine and Holland, 2018; Wenzel and Singh, 2018). Moreover, TK1 has been identified as a malignant biomarker in multiple malignancies due to its close correlation to cell proliferation, including lung, breast, and colorectal (Li et al., 2005; He et al., 2010; Nisman et al., 2014; Jagarlamudi et al., 2015; Weagel et al., 2018; McCartney et al., 2020). As for PCa, only several studies speculated that TK1 can be used as a diagnostic biomarker via bioinformatics analysis (Song et al., 2019; Wang et al., 2020). Song et al. integrated 10 eligible PCa microarray datasets via the Robust Rank Aggregation method and identified four candidate biomarkers, including TK1, for the diagnosis and prognosis of PCa (Song et al., 2019). Similarly, by informatic analysis of four PCa microarray datasets, Tian et al. identified six core genes including TK1 directly involved in the recurrence and prognosis of PCa (Wang et al., 2020). Although the above research has noted that TK1 is involved in PCa progression, experimental verification and potential mechanisms are still limited. In this study, the expression profile of TK1 was examined and results suggested that TK1 was up-regulated in PCa patients and cell lines, especially those with higher Gleason scores (> 7). We also identified the role of TK1 in PCa proliferation and migration via a series of experiments. In addition, 14 hub genes were identified via enrichment analysis and PPI network analysis, and their functions indicated that TK1 was closely involved in cell cycle-related signaling pathways, which was in accordance with the phenotypic results characterized previously. Moreover, we conducted the Kaplan-Meier survival analysis and Cox regression model and found that elevated TK1 expression was dramatically correlated with worse clinical survival.

The management of PCa still imposes an urgent challenge on society. Prostate specific antigen (PSA) screening has been performed for PCa diagnosis and relapse monitoring. But this could also lead to a series of problems such as overdiagnosis and overtreatment due to lack of specificity and poor indication of aggressiveness (Diamandis, 1998; Hayes and Barry, 2014). Therefore, new prognostic factor identification for biomedical recurrence and overall survival of PCa patients is crucial and urgent. Recently, many efforts have been made to find better biomarkers for PCa. Prostate-specific membrane antigen (PSMA), a type II transmembrane protein, has been found to be significantly overexpressed on prostatic cancer cells, including advanced-stage prostate carcinomas, but a low expression in normal tissues. It can be considered as ideal for developing small and low-molecular-weight targeted radiopharmaceuticals for diagnosis and treatment in imaging (Haberkorn et al., 2016). Therefore, it is more of a diagnostic and therapeutic target for imaging rather than a prognostic biomarker. Combined RankProd with genetic algorithm optimized artificial neural network (GA-ANN), Hou et al. identified a 15-gene signature that exhibited a great capacity for diagnosis and prognosis of PCa and found that C1QTNF3 was a good predictor for PCa diagnosis (Hou et al., 2018). However, the underlying mechanism lacks experimental validation, and more studies are warranted. Herein, we systemically demonstrated the function of TK1 in PCa and found that it can be applied as a prognostic biomarker. Similar to our results, much research has investigated the clinical value of serological TK1 in the diagnosis of PCa. Wang et al. determined the mean values and the concentration distribution of serological TK1 protein in a cohort of 56,178 persons consisting of people with different disease stages, and found that serological TK1 was a proliferating biomarker for early discovery of malignancy in the prostate (Wang et al., 2018). Jagarlamudi et al. demonstrated that there were inconsistencies in the particular activities as well as the subunit compositions of serological TK1 in different cancers. Meanwhile, serological TK1 protein assays can distinguish early-phase cancer formation in prostate and breast cancer more usefully than serological TK1 activity assays (Jagarlamudi et al., 2015). Furthermore, by collecting and analyzing serum samples from 140 patients, they also demonstrated that TK1 protein determinations together with Prostate Health Index (PHI) or PSA density (PSAD) can be worthy additional tools for PCa treatment (Jagarlamudi et al., 2019). However, the present study did not further determine the function of serological TK1 protein in PCa.

Tumor immune response plays a vital role in cancer formation and development. Though increasing evidence has proved the non-negligible role of immune system in PCa management, few approved immunotherapy exists (Bilusic et al., 2017; Cha et al., 2020). Using the TCGA database, Vesteinn Thorsson et al. classified tumors into six immune subtypes (Thorsson et al., 2019). Dramatic dissimilarities in lymphocyte infiltration, prognosis, and immune regulation gene expression existed among distinct subtypes. The present studies indicated that TK1 expression was dramatically decreased in C3 subtype of PCa, which had the best prognosis. This suggested that TK1 can be applied for immunophenotyping and prognosis prediction. The data from TISIDB also revealed that TK1 was significantly related with TILs and immunomodulators. Since the accumulation of TILs and immunomodulators expression in PCa was associated with patient prognosis, TK1 may be involved in immune tolerance via interacting with TILs and immunomodulatory molecules, and can be used as a potential marker for prostate immunotherapy (Steele et al., 2018; Pérez-Ruiz et al., 2020; Yang et al., 2021).

In conclusion, our research systematically explored the capacity of TK1 in PCa for the first time. Elevated expression of TK1 in PCa patients can be applied as a valuable prognostic biomarker for predicting poor survival (both DFS and OS). TK1 ablation inhibits tumor malignant behavior and may serve as a therapeutic target for PCa.

## Data Availability

The original contributions presented in the study are included in the article/Supplementary Material, further inquiries can be directed to the corresponding authors.

## Footnotes

1
https://www.proteinatlas.org/

2
http://cbioportal.org

3
https://metascape.org

4
https://cistrome.shinyapps.io/timer/

5
http://cis.hku.hk/TISIDB/index.php
