# Supplementary figures and images for: Assessing the Potential Prognostic and Immunological Role of TK1 in Prostate Cancer
Source: Front Genet. 2022 Apr 26;13:778850. doi: 10.3389/fgene.2022.778850 (PMC9086852; doi:10.3389/fgene.2022.778850)

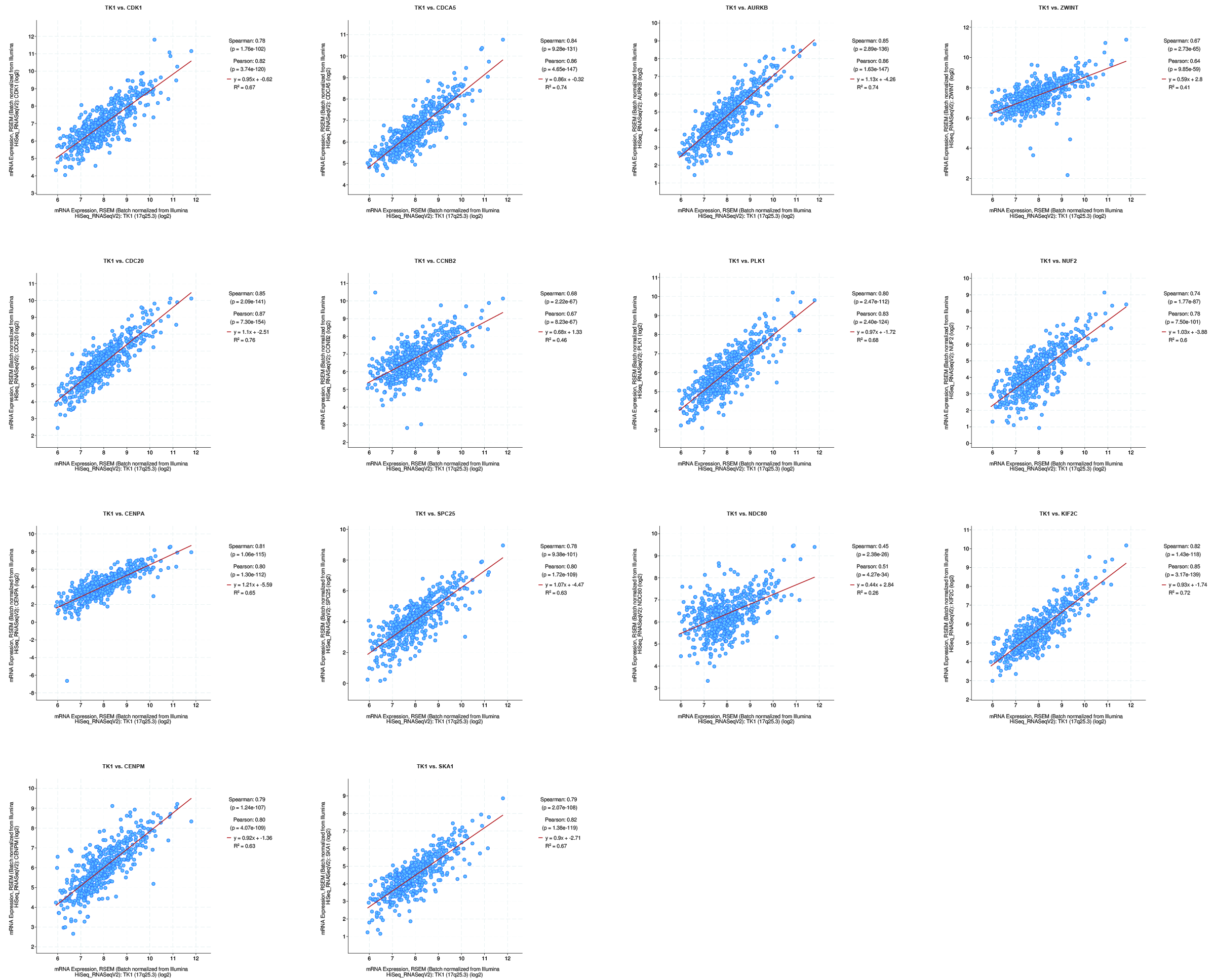

Supplement: Supplementary file 1 [file Image1.TIF]
