# Supplementary material for: Assessing the Potential Prognostic and Immunological Role of TK1 in Prostate Cancer
Source: Front Genet. 2022 Apr 26;13:778850. doi: 10.3389/fgene.2022.778850 (PMC9086852; doi:10.3389/fgene.2022.778850)
Supplement: Supplementary file 2 [file Table5.DOCX]

Table S1. The sequence of primers.

| Gene | Primer sequence |
| --- | --- |
| TK1 |  |
| Forward | ggggcagatccaggtgattc |
| Reverse | ccatggtgttccggtcatgt |
| AURKB |  |
| Forward | CAGTGGGACACCCGACATC |
| Reverse | GTACACGTTTCCAAACTTGCC |
| CCNB2 |  |
| Forward | CCGACGGTGTCCAGTGATTT |
| Reverse | TGTTGTTTTGGTGGGTTGAACT |
| CDC20 |  |
| Forward | GCACAGTTCGCGTTCGAGA |
| Reverse | CTGGATTTGCCAGGAGTTCGG |
| CDCA5 |  |
| Forward | GAGGTCCCAGCGGAAATCAG |
| Reverse | TCTTTAAGACGATGGGCTTTCTG |
| KIF2C |  |
| Forward | CAGAACTCTTACAGCTTCTTCCC |
| Reverse | CAGTGGACATGCGAGTGGA |
| CENPM |  |
| Forward | TGTGATGTCGGTGTTGAGGC |
| Reverse | GAGGGACTTTGCCAAGTGGA |
| GAPDH |  |
| Forward | TCGGAGTCAACGGATTTGGT |
| Reverse | TTCCCGTTCTCAGCCTTGAC |
| β-ACTIN |  |
| Forward | GGGAAATCGTGCGTGACATTAAG |
| Reverse | TGTGTTGGCGTACAGGTCTTTG |
